# Quantitative Analysis of Flavonoids and Coumarins from Fingered Citron in Different Growth Periods and Their Regulatory Effects on Oxidative Stress

**DOI:** 10.3390/foods14020180

**Published:** 2025-01-09

**Authors:** Tao Tan, Man Xu, Xianlong Hong, Zhenyuan Li, Jiangnan Li, Bining Jiao, Xijuan Zhao

**Affiliations:** 1 College of Horticulture and Landscape Architecture, Southwest University, Chongqing 400715, China; tantao123@email.swu.edu.cn (T.T.); 13658270352@163.com (M.X.); hxl1883@163.com (X.H.); zhenyuanl08@163.com (Z.L.); 2 Key Laboratory of Quality and Safety Control for Citrus Fruits, Ministry of Agriculture and Rural Affairs, Southwest University, Chongqing 400715, China; 13885084187@163.com (J.L.); jiaobining@cric.cn (B.J.)

**Keywords:** fingered citron, flavonoid, coumarin, RAW264.7 cells, oxidative stress

## Abstract

Twenty-two coumarins and twenty-six flavonoids were quantitated in fingered citron in different growth periods. Limettin was the top coumarin, and diosmin was the highest flavonoid, followed by hesperidin. Antioxidant evaluation by DPPH, ABTS, and FRAP indicated extracts of fingered citron in three growth periods all showed good antioxidant activity, which was positively correlated with the concentration of extracts. The oxidative stress model of RAW264.7 cells indicated extracts from fingered citron effectively reduced the contents of NO, MDA, and ROS in cells and increased the activity of SOD, thereby alleviating cell damage. The antioxidant capacity of fingered citron in November was the highest, followed by July and September. And there was a significantly positive correlation between the total flavonoid content and the antioxidant capacity. Diosmin, hesperidin, and neohesperidin were the main contributors to antioxidation. This study has significance for utilization of fingered citron germplasm resources and development of related functional products.

## 1. Introduction

Fingered citron (*Citrus medica* L. var. *sarcodactylis* Swingle) belongs to the Rutaceae family [1], a variety of citron and a kind of fruit, which can be eaten fresh or made into a dried product. It has a long history of dual use as food and medicine and has many pharmacological activities such as anti-inflammatory [2], antibacterial [3], and hypolipidemic effects [4]. Studies have shown that the chemical components of the fingered citron mainly include flavonoids, coumarins, organic acids, limonoids, and essential oils, which have significant contributions to its biological activities [3,5,6].

There has been some research on the identification and quantification of chemical components in fingered citron. Xia tentatively identified 67 components from dried powder of fingered citron, including 38 flavonoids and 29 coumarins [7]. Nogata et al. [8] and Ning et al. [9] quantified the contents of nobiletin, limettin, tangeretin, scopoletin, hesperidin, eriocitrin, rutin, diosmin, narirutin, and poncirin in fingered citron. Essential oils in fingered citron from different growth periods have been studied, and 27, 22, and 22 volatile components at the immature, middle, and mature stages were identified and quantified [10]. Results showed that the contents of monoterpenes and ketones accumulated gradually with fruit ripening, but the contents of sesquiterpenoids, esters, and other oxygen-containing derivatives showed a decreasing trend during fruit ripening. In addition, the immature stage showed the highest DPPH free radical scavenging ability.

Maturity is an important factor affecting the secondary metabolites in fruits, which will further affect the bioactivities of the fruit from the perspective of consumption. Moulehi et al. reported that for oranges, the highest content of both total flavonoids and phenols was found in the stage of commercial maturity, while for mandarins, the maximum content of total flavonoids and phenols was observed in the semimature and immature periods, respectively [11]. However, there is a lack of reports on the levels and changes in flavonoids and coumarins in fingered citron during fruit growth. In fact, fingered citron at different maturity levels has different applications. For example, green fruits can be used for flower arrangement and decoration, while yellow fruits are often used for medicine, tea, cooking, and Laoxianghuang [12,13,14,15]. Flavonoids and coumarins have been reported to show biological and pharmacological properties [1,16] and both of them are also important bioactive compounds in fingered citron. Investigation on the content changes of flavonoids and coumarins during different growth periods is of great significance for studying the efficacy of fingered citron at different maturity levels and the development of corresponding functional products. Previous studies mainly focused on non-targeted screening and identification of chemical components in fingered citron or quantitative analysis of some of the compounds with relatively high contents at one maturity level. The present research was carried out aiming to explore the levels and changes in flavonoids and coumarins in fingered citron at different growth periods and their correlation with the antioxidant capacity.

Oxidative stress refers to the breakdown of the balance between oxidation and anti-oxidation in the body and is accompanied by the accumulation of oxidative free radicals, resulting in abnormal cell structure and biological function, which is considered to be an important factor leading to aging and disease [17,18]. Natural antioxidants can effectively inhibit free radicals, so finding efficient and non-toxic natural antioxidants has become a hot topic in the field of scientific research. At present, two kinds of antioxidant evaluation methods are mainly used to evaluate the effect of antioxidants. Chemical methods such as DPPH, ABTS, and FRAP are relatively simple and easy and are suitable for preliminary screening and rough evaluation of a large number of samples and can provide preliminary information on antioxidant activity [19]. Using a cell model such as RAW264.7 cells is the other common antioxidant evaluation method. RAW264.7 cells contain various antioxidant enzymes such as superoxide dismutase (SOD) and can respond to oxidative stress. The secretion levels of reactive oxygen species (ROS), malondialdehyde (MDA), and nitric oxide (NO) are important indicators for evaluating antioxidant activity. The RAW264.7 cell model combined with chemical antioxidant evaluation method can evaluate the antioxidant ability of antioxidants scientifically and quickly.

In this study, targeted screening and quantitative analysis of flavonoids and coumarins in different growth periods of fingered citron were conducted, and the differences in their content and composition were compared. Meanwhile, the antioxidant activities of the extract of the fingered citron were investigated by chemical method combined with cell model, and the correlation between the main bioactive compounds of the fingered citron and their antioxidant activities was analyzed. This study will provide reference for the utilization of the fingered citron resources and the development of related functional products.

## 2. Materials and Methods

### 2.1. Materials and Chemicals

**Figure 1:**
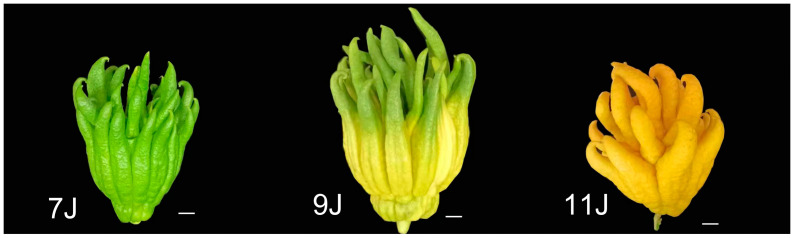
Fingered citron in 3 growth periods of July (7J), September (9J), and November (11J).

In this study, Jinhua fingered citron in three growth periods was taken as the experimental material, as shown in Figure 1. It was collected from Jinhua, Zhejiang Province, China, in July, September, and November, numbered 7J, 9J, and 11J, respectively. And the three growth periods were considered to be immature (green), semimature (only a part of the green turned yellow), and mature (golden yellow), respectively. Each growth period and origin of fingered citron was picked with the appropriate size, good development, and no pests and diseases of the fruit.

All chemical reagents were chromatographically pure except methanol, which was analytically pure. Methanol was purchased from Chengdu Chroma-Biotechnology Co., Ltd. (Chengdu, China); acetonitrile was purchased from Shanghai Titan Scientific Co.,Ltd. (Shanghai, China); and formic acid was purchased from Anpel Laboratory Technologies (Shanghai) Inc. (Shanghai, China). A total of 48 commercial standards were used in this study, including 22 coumarins and 26 flavonoids. The detailed information of the commercial standards such as name, CAS number, molecular formula, purity, and source is shown in Table S1 in the Supplementary Materials. Trolox, DPPH, and TPTZ were purchased from Shanghai yuanye Bio-Technology Co., Ltd. (Shanghai, China) and ABTS was from Xiya Reagent (Linyi, China). Materials and reagents used in RAW264.7 cell experiments, as well as the kits used for the determinations of SOD activity and secretion levels of ROS, MDA, and NO, are shown in Supplementary Materials Table S2.

### 2.2. Instruments

The instruments used in this study mainly include ultra-high-performance liquid chromatography/triple quadrupole tandem mass spectrometry with a turbo spray ion source (UPLC-QqQ-MS/MS, LCMS-8060NX010502, Shimadzu, Tokyo, Japan), chromatographic column (ACQUITY UPLC HSS T3, Waters, Milford, MA, USA), spectrophotometer (DU 730, Beckman Coulter, Brea, CA, USA), ultrapure water system double-evaporation water preparation instrument (Milli-Q Advantage A10, Millipore, Burlington, MA, USA), CO_2_ cell incubator (INC108, Memmert, Bremen, Germany), water bath (WB-20, SalvisLab, Zurich, Switzerland), autoclave (Hirayama HVE-50, HIRAYAMA, Saitama Prefecture, Japan), inverted phase contrast microscope (IX2-SL, OLYMPUS, Tokyo, Japan), clean workbench (SW-CJ-1F, Shanghai Boxun Industrial Co., Ltd. Shanghai, China), and multifunctional enzyme marker (VARIOSKAN LUX, Thermo Scientific, Waltham, MA, USA).

### 2.3. Sample Preparation

#### 2.3.1. Pretreatment

The fingered citron was washed with clean water. After being dried and cut into thin strips, they were put into the oven to dry by setting the temperature at 50 °C. And then, the dried products were pulverized with a powder grinder and screened with a 40-mesh sieve. The obtained powder was stored in a freezer at −20 °C for further use.

#### 2.3.2. Extraction

The extraction was according to the method reported previously by our group with some modifications [20]. Sample powder of 0.5 g was accurately weighed and put into 10 mL centrifuge tubes. Methanol (7 mL) was added into each tube to ensure that the sample powder and methanol were fully mixed. The mixture was extracted under ultrasonication at 300 W at room temperature for 30 min and then centrifuged. The centrifuge conditions were set to 10 min and 10,000 r/min. After centrifugation, the supernatant was taken and placed in a 50 mL centrifuge tube, and then 7 mL methanol was added to the residue. The above operation was repeated two times, and the supernatant obtained from three replicates was combined. Finally, the volume was fixed to 25 mL using methanol. The extract was stored in refrigerator at 4 °C for use later.

#### 2.3.3. Concentration, Filtration, and Dilution

Methanolic extract (3 mL) was divided using 4 mL centrifugal tubes and centrifuged at room temperature by vacuum centrifugation at 2000 r/min. After the concentration was completed, the obtained sample was diluted to 1 mL with ultrapure water (18.2 MΩ) and filtered into a vial with a syringe filter with 0.22 μm PTFE membranes for further detection.

Due to the significant differences in the content of various flavonoids and coumarins in the real samples, each sample extract was diluted by different quantities. After that, both the original and the diluted samples were detected in order to ensure that the content of each analyte is within the linear range of the corresponding standard curve and to ensure the accuracy of the quantitative results.

### 2.4. UPLC-QqQ-MS/MS Conditions

The column temperature was 40 °C. The flow rate was 0.4 mL/min. The injection volume was 1.0 μL. Scheduled multiple reaction monitoring (sMRM) was used with switching of positive and negative ion mode for data collection. The mobile phase consisted of (A) water containing 0.10% formic acid and (B) acetonitrile, and the elution procedure was 0~0.5 min: 10% B; 0.5~6.0 min: 10% B~35% B; 6.0~8.0 min: 35% B~70% B; 8.0~9.5 min: 70% B~70% B; 9.5~11 min: 70% B~100% B; 11.0~13.0 min: 100% B~100% B; 13.0~13.01 min: 100% B~10% B; 13.01~15.0 min: 10% B. Detailed MRM instrument parameters are shown in Table S3 in the Supplementary Materials such as ion types, precursor ions, product ions, collision energy (CE) of each product ion, desolvation temperature, gas flows, some voltages, and so on.

### 2.5. Method Validation

A series of mixed standard solutions of 0.1, 1, 5, 10, 25, 50, 100, 200, 300, 400 μg/L were obtained by diluting the mixed stock solution of the commercial standards, which were used for linear evaluation of the analytical method for the 48 compounds. At least 5 of the 10 concentrations were used and their corresponding peak areas of the quantitative ion were linearly fitted to obtain calibration curves. The sensitivity of the method is expressed by the limit of detection (LOD) and the limit of quantitation (LOQ), which are the concentrations of the corresponding compound at the S/N ≥ 3 and S/N ≥ 10, respectively. Accuracy was indicated by the recovery rates. Adding 48 mixed standard solutions with concentrations of 50 μg/L and 100 μg/L to the extract of the fingered citron, the concentration difference of each target compound before and after the addition of the commercial standards should be determined. The recovery rate of each compound was the ratio of concentration difference of the corresponding compound to the known added amount.

### 2.6. Evaluation Method of Antioxidant Activity

#### 2.6.1. Chemical Methods

The determination of DPPH free radical scavenging ability was slightly modified from the method of Gorinstein et al. [21], which is briefly described as follows. Trolox standard solution or sample extraction solution of 0.5 mL at different concentrations was mixed with 3.5 mL 75 μmol/L DPPH solution, and the reaction was performed for 30 min in the dark. At the same time, 0.5 mL 80% methanol solution was mixed with 3.5mL DPPH solution as a control. After the reaction, the absorbance of each sample was measured at 517 nm, and the antioxidant capacity was expressed by Trolox equivalent TE (μmol/L).

The determination of free radical scavenging ability of ABTS referred to Almeida et al. [22] with slight modification, which is briefly shown as follows. K_2_S_2_O_8_ solution (140 mmol/L; 8 μL) was mixed with 5 mL of 7 mmol/L ABTS, and the mixed solution reacted in the dark for 12–16 h to completely oxidize ABTS to ABTS cationic free radicals (ABTS^•+^). The solution of ABTS cationic radical was diluted with anhydrous ethanol until its absorbance at 732 nm was 0.70 ± 0.02. Trolox standard solution or sample extract solution of 0.1 mL at different concentrations was mixed with 3.9 mL ABTS^•+^ solution, and 0.1 mL 80% methanol solution was mixed with 3.9 mL ABTS cationic radical solution as the control, and the reaction was carried out for 10 min in the dark. The absorbance of each sample was measured at 734 nm, and the antioxidant capacity was expressed by Trolox equivalent TE (μmol/L).

The slightly modified method of Benzie et al. [23] was used for the determination of the iron ion reduction capacity of FRAP. Acetic acid buffer (200 mL, 0.3 mol/L), TPTZ working solution (20 mL, 10 mmol/L), and FeCl_3_ solution (20 mL, 20 mmol/L) were mixed to make TPTZ working solution. Trolox standard solution or sample extraction solution (0.2 mL) at different concentrations were taken to mix with 3.8 mL TPTZ working solution. At the same time, the same volume of 80% methanol solution was mixed with TPTZ working solution as a control. After the reaction in the dark, the absorbance of each sample was measured at 593 nm. Trolox equivalent TE (μmol/L) was used to indicate the antioxidant capacity.

#### 2.6.2. RAW264.7 Cell Model

A 6-well culture plate was used, and 45 mL high-glucose DMEM medium, 5 mL FBS, and 1 mL double-antibody medium were successively added into the 50 mL centrifuge tube to form complete medium. When the density of RAW264.7 cells reached about 80%, the old culture medium in the cell plate was discarded, washed twice with 1 mL PBS, and then, 2 mL of the newly complete medium was added. The RAW264.7 cells attached to the wall were gently scraped off with a cell scraper, gently blown into cell suspension with a pipette, and then the cell suspension was sucked into a 50 mL centrifuge tube. After centrifugation at 1000 r/min for 5 min, the suspension was discarded and 2 mL of complete medium was added to the centrifuge tube, which was further transferred to a new cell culture plate, shaken to make it spread evenly, and cultured in an incubator containing 5% CO_2_ at 37 °C.

RAW264.7 cells were used to evaluate the regulatory effect of different concentrations of fingered citron extract on cell survival in the absence and presence of oxidative stress effects induced by H_2_O_2_. The concentration of RAW264.7 cells in the logarithmic growth stage was adjusted to 1 × 10^5^ cells/mL, and 100 μL per well was inoculated into the 96-well plate with 5 parallel wells for each component. The cells were cultured at 37 °C and 5% CO_2_ for 24 h to ensure good adhesion. The original medium was discarded and the cells were treated with 100 μL H_2_O_2_ or fingered citron extract at different concentrations. Simultaneously, a control and a blank were set up. After 24 h of cell culture, a newly prepared complete medium containing 10% CCK-8 reagent was added to each well instead of the original medium. The cells continued to grow during the incubation period of 1–4 h, and the OD value of the cells was accurately measured at 450 nm. The relative cell survival rate was calculated using Formula (1).

(1) $$Therelativecellsurvivalrate\left(\%\right)=\frac{\left[OD\left(experimentalgroup\right)-OD\left(blankgroup\right)\right]}{\left[OD\left(controlgroup\right)-OD\left(blankgroup\right)\right]}$$

The OD (experimental group) indicated the OD values of wells containing cells with H_2_O_2_ and CCK-8 solution added. The OD (blank group) meant the OD values of wells containing no cells with CCK-8 solution but without H_2_O_2_. The OD (control group) indicated the OD value of pores containing cells and CCK-8 solution without H_2_O_2_.

The concentration of RAW264.7 cells in logarithmic growth stage was adjusted to 5 × 10^5^ cells /mL, and the cells were inoculated in 96-well plates with 100 μL per well and cultured overnight at 37 °C. The cells were divided into control group, model group, and experimental group. The experimental group was treated with the final concentration of 400 mg/L, 600 mg/L, and 1000 mg/L of fingered citron extract. Except the normal control group, the other groups were treated with a complete culture medium containing 0.004% H_2_O_2_. After 2 h, the culture medium was discarded and the cells were washed twice with PBS, and then fresh medium was directly added to the model group. After 24 h, cell viability, SOD activity, and secretion levels of ROS, MDA, and NO were measured.

NO secretion level was measured using the Griess kit and can be described briefly below. NaNO_2_ solution of 1 mmol/L was diluted to a series of standard solutions of different concentrations, and 100 μL was taken from each concentration of standard solution and was mixed with an equal amount of Griess reagent. The mixture further reacted in the dark for 10 min at room temperature. After that, the absorbance of each sample at 550 nm was measured. Then, a linear equation was obtained according to the concentration of NO and the corresponding absorbance. Similarly, 100 μL cell supernatant was taken to react with Griess reagent and the absorbance at 550 nm was measured according to the above method. The NO content was calculated based on the linear equation.

ROS secretion level was measured using an ROS detection kit, which could be briefly described as follows. Cells were collected in the centrifuge tube. By centrifugation, the cells were separated from the supernatant, which was discarded subsequently. The diluted DCFH-DA solution of 1 mL as the fluorescent probe was added to the centrifuge tube to make the cells and DCFH-DA mix fully. After incubation for 20 min, the cells were washed three times in order to effectively remove the unreacted DCFH-DA. Finally, the fluorescence intensity of the cells was measured with the excitation wavelength of 488 nm and the emission wavelength of 525 nm, which can reflect the level of intracellular ROS.

The content of MDA was determined according to the MDA content detection kit. A brief description is shown below. The cells were collected and centrifuged to discard the supernatant. The extraction solution of 1 mL was added to the centrifuge tube to extract MDA from cells. In order to crush cells more effectively, an ultrasonic treatment was used at a power of 200 W for 3 s with an interval of 10 s. The same procedure was repeated 30 times to ensure that the cells were fully fragmented. After completing the ultrasonic crushing, centrifugation was carried out again at 8000 r/min for 10 min at 4 °C to ensure that MDA was fully released into the extraction solution. The supernatant was taken for further processing and subsequent absorbance measurement at 532 nm and 600 nm according to the instructions of the MDA content detection kit. Finally, the content of MDA was calculated based on the difference in absorbance changes of the sample at 532 nm and 600 nm.

The measurement of SOD activity used an SOD activity test kit and a brief description is as follows. The RAW264.7 cells were collected and centrifuged to discard the supernatant. The extraction solution (1 mL) was added to the remaining cells with an ultrasonic treatment to make the cells fully fragmented. The mixture was centrifuged again at 8000 r/min for 10 min at 4 °C. The supernatant was obtained and put on ice for further determination. According to the instructions of the kit, the mixed solution was prepared. After incubation for 30 min at 37 °C, the absorbance of the sample in each tube was measured at 560 nm to evaluate the SOD activity.

### 2.7. The Antioxidant Potency Composite (APC) Index

The APC index is a comprehensive indicator for evaluating antioxidant activity of samples. It combines the results of different testing methods for antioxidants to provide a more comprehensive assessment of antioxidant capacity [24]. The calculation method of the APC index in this study is shown in Formula (2):

(2) $$A\mathrm{PC}\;(\%)=\sum_{k=1}^{N}\left\{\frac{{\left[\sum_{i=1}^{n}\left(\frac{C_{i}}{C_{max}\times n}\right)\times 100\right]}_{k}}{N}\right\}\;$$

where C is the antioxidant value of each evaluation method at the same concentration, C*_max_* is the maximum antioxidant value of the evaluation method at the same concentration, *n* indicates three chemical evaluation methods of antioxidant capacity, and *N* represents five concentrations of the samples.

### 2.8. Data Analysis

LabSolutions software (version 5.99) was used to perform the linear fitting of 48 commercial standards and the quantitative analysis of all samples, including the recovery experiments, as well as to obtain the LOD and LOQ. SkanIt RE4.1 software was used for absorbance detection and data collection. IBM SPSS Statistics 26 was used for significance testing, and Origin 2021 was used to draw bar charts, chromatograms, and heatmaps for correlation analysis.

## 3. Results and Discussion

### 3.1. Analysis of Flavonoids and Coumarins in Fingered Citron Based on UPLC-QqQ-MS/MS

#### 3.1.1. Method Establishment and Validation

**Figure 2:**
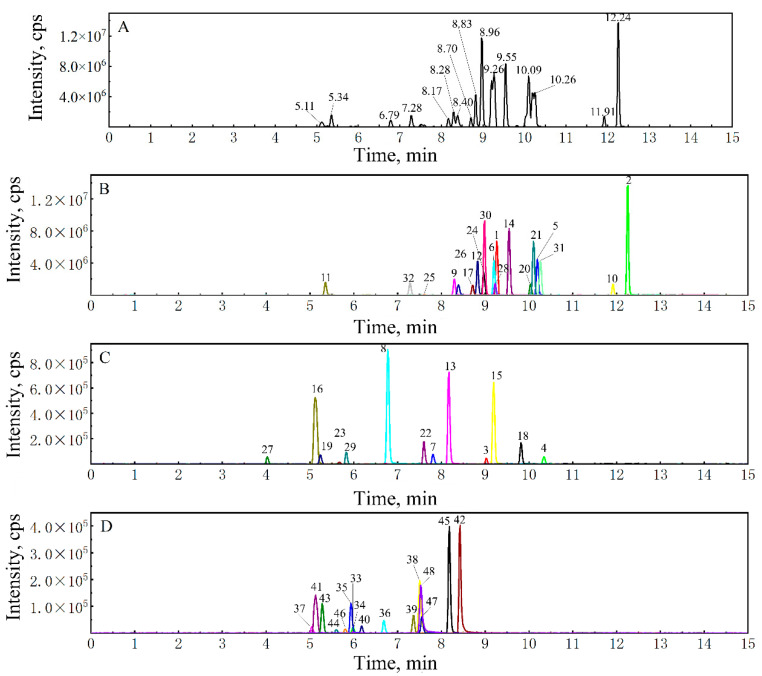
UHPLC-MS chromatograms. (**A**) The total ion chromatograms of 48 mixed standards. (**B**,**C**) The extracted ion chromatograms of sMRM traces from 32 mixed standards in the positive ion mode. (**D**) The extracted ion chromatogram of sMRM from 16 mixed standards in the negative ion mode. Compounds corresponding to serial numbers in the figure are shown in the Supplementary Materials, Table S4.

A quantitative analysis method for 22 coumarins and 26 flavonoids was established. MRM parameters for each compound (displayed in Supplementary Materials, Table S3) were optimized based on their respective commercial standards and previous research [20,25] in order to find the product ions and their optimal collision energy for qualitative and quantitative analysis. This method utilized both positive and negative ion modes simultaneously. There are eight isomers in the 48 compounds, which are scoparone and limettin, imperatorin and isoimperatorin, tangeretin and sinensetin, vitexin and isovitexin, didymin and poncirin, cnidilin and phellopterin, hesperidin and neohesperidin, and naringin and narirutin. Despite each pair of isomers having the same molecular weight and similar chemical structures, they could be successfully distinguished by the precise ion pair matching and retention time analysis. In the extraction ion chromatograms (Figure 2), the good separation of the standard mixture of these 48 compounds can be clearly observed. Most flavonoids and coumarins showed strong signals in the positive ion mode, so there were more peaks in the positive ion mode, and fewer peaks in the negative ion mode.

In order to verify whether this method can be applied to the detection of real samples, the linear relationship, sensitivity, and accuracy were evaluated. A series of mixed standard solutions for 48 compounds was used for linear evaluation. The linear correlation coefficients for all compounds were greater than 0.99 (Supplementary Materials, Table S4), indicating an excellent linear relationship between the concentration of each compound and the peak area of its quantitative ion pair. Under this method, the sensitivity of 48 compounds was different with LODs of 39 compounds ≤ 1.00 μg/L and LOQs of 32 compounds ≤ 1.00 μg/L. Among them, this method showed the highest sensitivity for vitexicarpin with an LOD and LOQ of 0.01 μg/L and 0.02 μg/L, respectively. The recoveries ranged from 76.23% to 123.53% when the added amount was 50 μg/L and from 80.77% to 125.91% when the added level was 100 μg/L. The results indicate that the accuracy of the method was satisfactory and can meet the requirements of detection. Overall, this quantitative method based on UPLC-QqQ-MS/MS was shown to have excellent sensitivity and accuracy. Therefore, it can be used for the precise screening and quantitative analysis of flavonoids and coumarins in fingered citron samples.

#### 3.1.2. Targeted Screening of Flavonoids and Coumarins in Fingered Citron

**Figure 3:**
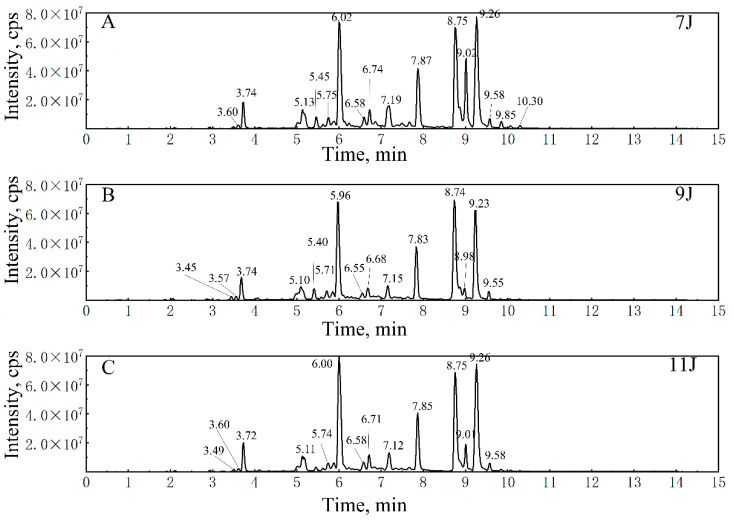
Total ion chromatograms of the extract of fingered citron in July (**A**), September (**B**), and November (**C**).

This validated method was further used for targeted screening and quantitative analysis of flavonoids and coumarins in fingered citron from three growth periods (Figure 3), aiming to reveal the content changes and distribution rules of these compounds in fingered citron.

A total of 21 flavonoids and 21 coumarins were detected in 7J and 9J, and 21 flavonoids and 19 coumarins were detected in 11J (Supplementary Materials, Table S5). Quercetin, isorhamnetin, rhoifolin, phloretin, poncirin, and psoralen were not detected in the fingered citron for three growth periods. 4′,5,6,7-tetramethoxyflavone and osthole were detected in the three growth periods but could not be accurately quantified using the linear equation of the method (Supplementary Materials, Table S4) owing to the very low contents. 5-Geranyloxy-7-methoxycoumarin was not detected in 11J, but could be quantified both in 7J and 9J, showing that as the maturity period approached, its content gradually decreased. 6′,7′-dihydroxybergamottin was detected in 7J but could not be accurately quantified. Combined with the contents in 9J and 11J, it was observed that the content of 6′,7′-dihydroxybergamottin gradually increased as the fruit matured. 6′,7′-epoxybergamottin was detected in 9J but could not be accurately quantified and was not detected in 11J. In general, the types of flavonoids and coumarins in different growth periods were basically the same. A total of 42 compounds were detected in all samples, among which 41 compounds were accurately quantified. This is a comprehensive study on the quantitative analysis of flavonoids and coumarins in fingered citron fruit.

#### 3.1.3. Analysis of the Contents of Flavonoids and Coumarins in Fingered Citron

**Figure 4:**
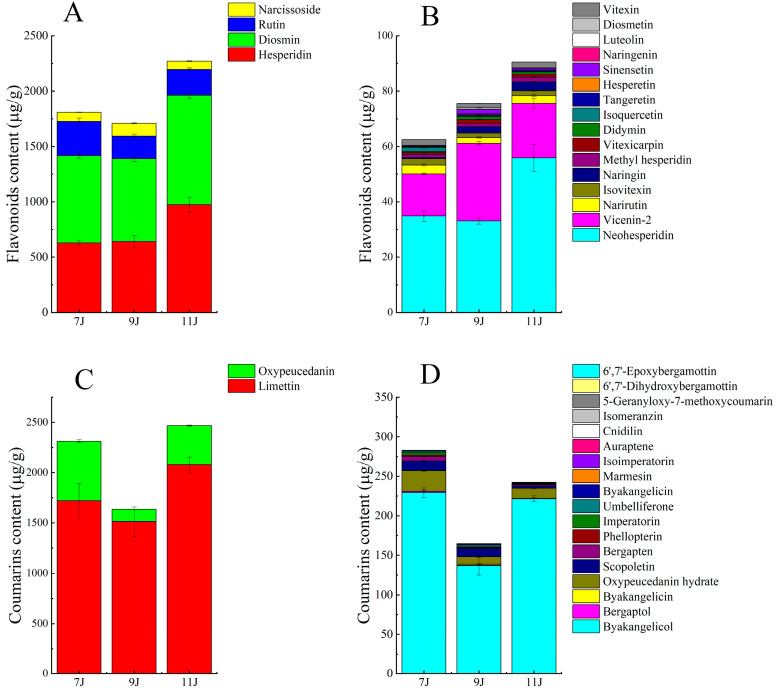
Contents of flavonoids and coumarins in the fingered citron of July (7J), September (9J), and November (11J). (**A**,**B**) The contents of flavonoids. (**C**,**D**) The contents of coumarins.

As the fruit grew and developed, the contents of total flavonoids detected here in fingered citron first decreased slightly from July to September and then increased to the highest level of 2360.89 ± 123.753 μg/g in November (Figure 4). The content of diosmin was always the highest among all these flavonoids in the three growth periods, indicating the importance of diosmin in the fingered citron. Followed by hesperidin, its content was more than 500 μg/g. The content of rutin in the three growth periods is higher than 100 μg/g, and that of narcissoside, neohesperidin, and vicenin-2 content was higher than 10 μg/g. For coumarins, the total content first decreased and then increased, showing the same trend with flavonoids. And the content of total coumarins was also the highest in November in terms of the three growth periods, in which limettin dominated accounting for 66.34–84.00% of the total content of coumarins. Followed by oxypeucedanin and byakangelicol, each of them had the contents more than 100 μg/g. Besides diosmin and limettin, both hesperidin and neohesperidin also showed the highest contents in 11J compared with those in 7J and 9J.

It has been reported that some flavonoids and coumarins were quantified in fingered citron at the stage of maturity. Chu et al. detected that the contents of diosmin and hesperidin in Guang fingered citron were 1204.8 ± 1.4 μg/g and 801.6 ± 18.8 μg/g, respectively [26]. Limettin was 779.0 ± 7.3 μg/g, which was higher than scopolamine 34.2 ± 0.3 μg/g, scoparone 15.5 ± 0.2 μg/g, and bergapten 1.7 ± 0.0 μg/g. Xia et al. also pointed out that the content of limettin in the five samples from different origins ranked first, among which the content of limettin in the sample from Sichuan was as high as 1609.2 ± 65.1 μg/g [7]. Ning et al. showed that the flavonoid with the highest contents in fingered citron was hesperidin, and the coumarin with the highest content was limettin [9], which is almost consistent with the results of this study. However, Wu determined by HPLC that the content of rhoifolin in Zhejiang fingered citron was as high as 315.75 μg/g and that the content of scoparone in Sichuan fingered citron was as high as 364.83 μg/g [27]. Cui also determined by HPLC that the contents of scoparone and scopoletin in 48 Sichuan fingered citron were mostly above 100 μg/g [28], while in this study, the contents of rhoifolin, scoparone and scopoletin were below 10 μg/g. Fingered citron is rich in bioactive compounds and there are several groups of isomers in the extract. So, the detection methods, sample sources and pretreatment steps, etc. may be the factors that lead to the differences in the final quantitative results of flavonoids and coumarins at the same growth period of fingered citron.

From the perspective of different growth periods, there is currently a lack of relevant reports on the levels of the flavonoids and coumarins in fingered citron. Results in this study indicated that the content of some compounds such as hesperidin gradually increased as the fruit matured and some other compounds showed different trends. For example, the content of limettin first decreased and then increased. The biosynthesis of flavonoids and coumarins is a complex regulatory process involving the expression of multiple enzymes and genes. At different stages of growth and development, the expression levels of some enzymes and genes will change, thereby affecting the synthesis and accumulation of flavonoids and coumarins. Also, different growth stages may activate or inhibit specific metabolic pathways, leading to changes in the content of a certain flavonoid or coumarin. Meanwhile, environmental stress such as light, temperature, water, etc., are possible factors that can affect the activity of flavonoid and coumarin synthase, which in turn affects the accumulation of flavonoids and coumarins.

### 3.2. Evaluation of Chemical Antioxidant Activity of Fingered Citron Extracts

Three methods were used to evaluate the in vitro chemical antioxidant capacity of the fingered citron extract in three different growth periods. With the concentration increase in the extract, the scavenging effect on DPPH free radicals and ABTS cationic free radicals also gradually increased. Through the determination of the iron ion reduction capacity of FRAP, it was observed that these extracts could reduce iron ions more effectively at high concentrations, further demonstrating their antioxidant effects. The results obtained by the three methods were different to some extent, but the overall trend was the same. The APC index is known to be a comprehensive indicator for evaluating the antioxidant activity of samples. The results of the APC index analysis of the antioxidant activity of the fingered citron extract are shown in Table 1. The APC index of 11J extract was the highest, at 100%, and the scavenging ability on DPPH free radicals and ABTS free radicals, as well as the reduction ability of the FRAP iron ion, were the highest among the three in vitro antioxidant tests. Finally, APC comprehensive ranking was used to determine the order of antioxidant capacity in each growth period. In general, the fingered citron picked in November showed the strongest antioxidant activity, followed by that in July.

### 3.3. Effects of Fingered Citron Extract on Oxidative Stress of RAW264.7 Cells Induced by H_2_O_2_

#### 3.3.1. Effects of Fingered Citron Extract on the Survival Rate of RAW264.7 Cells

The fingered citron extract with high concentrations probably inhibited the growth of RAW264.7 cells. Through cytotoxicity detection, the optimal concentration range of fingered citron extract can be determined. The final concentrations of 300 mg/L, 600 mg/L, 1000 mg/L, 3000 mg/L, and 6000 mg/L were added to the cell culture, and the cell viability was detected after 24 h. When the concentration of fingered citron extract in the three growth periods was 0~1000 mg/L, the cell survival rate had no significant change (*p* > 0.05) (Supplementary Materials, Figure S1). When the concentration of fingered citron extract was 3000 mg/L, the cell viability of 11J and 9J showed no significant difference (*p* > 0.05) compared to the control group, while the cell survival rate of 7J was only 75.30%, which was significantly lower than that of the control group (*p* < 0.05), indicating that this concentration had some toxicity to cells. When the concentration of the extract in the three growth periods was 6000 mg/L, the survival rates of 7J, 9J, and 11J were only 14.18%, 86.43%, and 71.07%, respectively, indicating that 6000 mg/L of extract was not conducive to cell growth. Therefore, the concentrations of 300 mg/L, 600 mg/L, and 1000 mg/L were selected as the low, medium, and high dose groups of fingered citron extract for subsequent studies, respectively.

#### 3.3.2. Optimization of H_2_O_2_ Concentration

The cells were observed under a microscope and photographed (Supplementary Materials, Figure S2). When there was no H_2_O_2_, the cells were round and plump with a large number and strong adhesion to the wall. When different concentrations of H_2_O_2_ were added, the cells were gradually damaged. The cell edges were no longer round and began to curl toward the middle with gradually larger cell spacing. When the concentration of H_2_O_2_ was greater than 0.008%, there was hardly any cell survival under the microscope, indicating that the cells were strongly damaged, with particularly low cell survival rates.

Compared with the blank group, different concentrations of H_2_O_2_ treatment significantly reduced the survival rate of RAW264.7 cells (*p* < 0.05), which demonstrated that different concentrations of H_2_O_2_ had a significant inhibitory effect on the growth of RAW264.7 cells, and this inhibitory effect was positively correlated with the increase in H_2_O_2_ concentration. When RAW264.7 cells were treated with 0.004% H_2_O_2_, the survival rate of RAW264.7 cells was 49.93% (Supplementary Materials, Figure S3). This suggests that 0.004% H_2_O_2_ could cause significant damage to the cells but would not lead to a large number of cell deaths. When the concentration of H_2_O_2_ was low, the damage to RAW264.7 cells was not significant. With the increase in H_2_O_2_ concentration, the toxic effect on cells was gradually enhanced, and when the concentration of H_2_O_2_ was too high, a large number of cells would die, which was adverse to the subsequent research. So the oxidative stress model induced by H_2_O_2_ was established using 0.004% H_2_O_2_.

#### 3.3.3. The Regulatory Effects of Fingered Citron Extract on Oxidative Stress of RAW264.7 Cells Induced by H_2_O_2_

Compared with the control group, the activity of RAW264.7 cells in the H_2_O_2_ model group was significantly decreased (*p* < 0.05) (Supplementary Materials, Figure S4), indicating that 0.004% H_2_O_2_ had a significant inhibitory effect on the growth and proliferation of RAW264.7 cells, while pretreatment with 400 mg/L, 600 mg/L, and 1000 mg/L fingered citron extract could significantly relieve the inhibitory effects of H_2_O_2_ on the activity of RAW264.7 cells (*p* < 0.05), and the cell activity increased with the increase in extract concentration, indicating that fingered citron extract had a protective effect on cells. Also, it was observed that the extract of 11J showed a strong protective effect on H_2_O_2_-induced oxidative stress injury, especially in the high-dose group with the cell survival rate of 90.17%. Such a result was consistent with the in vitro antioxidant activity tested by DPPH, ABTS, and FRAP, which also showed that the 11J had the highest antioxidant activity.

#### 3.3.4. Effects of Fingered Citron Extract on NO Content of RAW264.7 Cells Induced by H_2_O_2_

**Figure 5:**
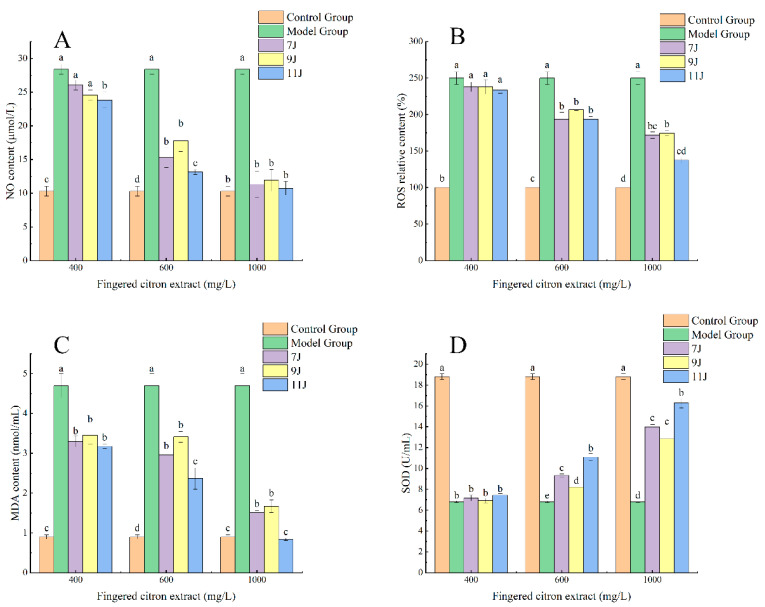
Effects of fingered citron extract on the contents of NO, ROS, MDA, and SOD in RAW264.7 cells after H_2_O_2_ treatment. (**A**–**D**) The contents of NO, ROS, MDA, and SOD under different concentrations of fingered citron extract, respectively. Different lowercase letters in the histograms represent significant differences between groups (*p* < 0.05) and the same lowercase letters indicate no significant difference.

NO can be generated during L-arginine synthesis catalyzed by NO synthase. It plays a complex and diverse role in organisms, not only participating in the transmission of information between cells but also playing a key role in inflammation and immunity. However, it may be overproduced under oxidative stress, leading to cell damage [29]. Sodium nitrite was diluted into a series of standard solutions with the concentrations of 1, 2, 5, 10, 20, 40, 50, 60, 80, 90, 100 μmol/L. The corresponding absorbance was measured with Griess reagent after color rendering, and the calibration curve was drawn between absorbance and the NO concentration, which was *A* = 0.0066 *c*_NO_ + 0.1172. The linear relationship was excellent with *R ^2^* > 0.99 (which can be used for the determination of NO content. Compared with the control group, the NO level in cell culture medium in the model group was significantly increased (*p* < 0.05). Compared with the model group, except for the low dose group, the level of NO in the cell culture medium of the group treated with fingered citron extract was significantly decreased (*p* < 0.05). There was a significant difference in the NO contents between the high dose group and the control group (*p* > 0.05), indicating that the high dose of the extract had a significant regulatory effect on NO secretion of the RAW264.7 cells stimulated by H_2_O_2_ (Figure 5A).

#### 3.3.5. Effects of Fingered Citron Extract on ROS Content of RAW264.7 Cells Induced by H_2_O_2_

ROS play an important role in normal physiological conditions and participate in a variety of cell signaling processes. However, when ROS are overproduced, they can cause serious damage to organisms. ROS show high oxidizability and can attack biological molecules such as DNA and proteins resulting in their structural damage. More seriously, ROS can hinder the repair of damaged DNA, making it difficult for cells to return to normal function [30,31]. As shown in Figure 5B, ROS levels in cells of the H_2_O_2_ model group were significantly increased compared with the control group (*p* < 0.05), which indicated that 0.004% H_2_O_2_ could significantly promote the vast production of free radicals in RAW264.7 cells, thus triggering oxidative stress reactions. Treatment with different concentrations of fingered citron extract could inhibit the rise of ROS levels caused by H_2_O_2_. When the fingered citron extract was used in medium or high doses, the ROS levels were significantly lower than those in the H_2_O_2_ model group (*p* < 0.05), indicating that the fingered citron extract could effectively inhibit H_2_O_2_-induced ROS production. When the extract of 11J was used in the high dose of 1000 mg/L, there was no significant difference of the ROS levels between 11J and the control group (*p* > 0.05). The results showed that the 11J extract of 1000 mg/L could restore the ROS level to near the normal value. At the same dose, the ability of 7J and 9J to scavenge ROS was much weaker than that of 11J.

#### 3.3.6. Effects of Fingered Citron Extract on MDA Content in RAW264.7 Cells Induced by H_2_O_2_

MDA is the product of lipid peroxidation of unsaturated fatty acids, and its accumulation in the body can cause damage to cell and tissue. It is often used to reflect the level of lipid peroxidation [32]. The increased content of MDA usually means that cells have suffered oxidative damage. Compared with the control group, MDA content in cell culture medium of the H_2_O_2_-induced model group was significantly increased (*p* < 0.05), indicating that H_2_O_2_ did lead to cellular oxidative stress. Compared with the model group, the low, medium, and high dose groups significantly inhibited the increase in MDA content caused by H_2_O_2_ (*p* < 0.05) and the inhibition was dose-dependent. This means that the extract of fingered citron has an antioxidant effect, which can reduce the oxidative damage of cells caused by H_2_O_2_, and this antioxidant effect is enhanced with the increase in the amount of fingered citron. In the high-dose group, the MDA content of 11J was 0.833 ± 0.027 nmol/mL, which was close to the level of the control group with no significant difference between them (*p* > 0.05). This showed that at high doses, the fingered citron extract almost completely inhibited the oxidative stress response of RAW264.7 cells induced by H_2_O_2_, and the MDA level returned to nearly normal (Figure 5C).

#### 3.3.7. Effects of Fingered Citron Extract on SOD Activity of RAW264.7 Cells Induced by H_2_O_2_

SOD is an important enzyme that regulates oxidative stress in the body. SOD can convert O_2_^-^· into H_2_O_2_, and the level of SOD activity can reflect the ability of cells to clear free radicals [33]. SOD activity in the normal control group was 18.80 ± 0.27 U/mL and decreased to 6.80 ± 0.079 U/mL after adding 0.004% H_2_O_2_, indicating that H_2_O_2_ had a significant effect on SOD activity. SOD activity in the cell culture medium of the model group was significantly decreased (*p* < 0.05), which further confirmed the inhibitory effect of H_2_O_2_ on SOD activity. Compared with the model group, the SOD activity of fingered citron extract in the low-dose group did not increase significantly in the three growth periods (*p* > 0.05), and the other groups were significantly higher than the model group (*p* < 0.05). Furthermore, the change in SOD activity in the cell supernatant showed a significant difference related to the concentration of fingered citron in a dose-dependent manner (Figure 5D).

### 3.4. Correlation Analysis of Bioactive Compounds and Antioxidant Capacity

According to the results of previous studies, hesperidin, diosmin, rutin, neohesperidin, and narcissoside are important flavonoids in fingered citron, while limettin, oxypeucedanin, byakangelicol, bergapten, scoparone, and scopoletin are important coumarins in fingered citron. The antioxidant capacity of fingered citron extract at different growth periods was determined by four different methods, namely DPPH, ABTS, FRAP, and cellular antioxidant evaluation (CAA). Here, the correlation between these four antioxidant test methods and the main bioactive compounds of fingered citron was analyzed.

**Figure 6:**
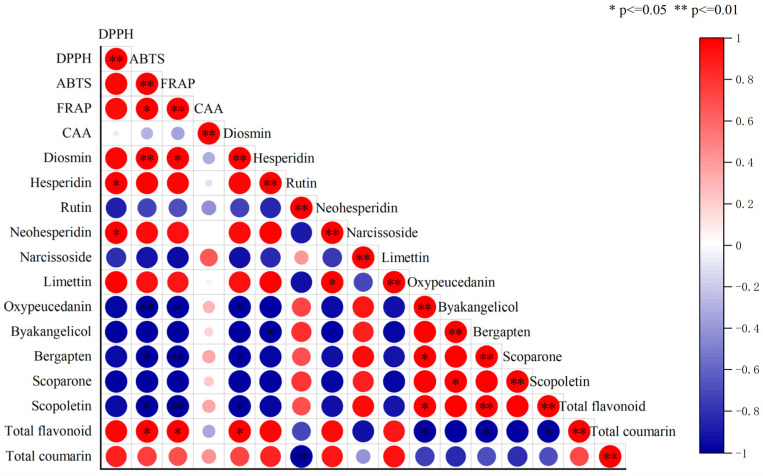
Correlation analysis of flavonoids and coumarins with antioxidant capacity.

As shown in Figure 6, the three chemical methods showed strong correlation, and ABTS was significantly positively correlated with FRAP (*p* < 0.05). There was no statistical correlation between the results of CAA and the three chemical methods of DPPH, ABTS, and FRAP (*p* > 0.05). The content of diosmin was significantly positively correlated with the result obtained by the ABTS (*p* < 0.01) method and significantly positively correlated with the FRAP result (*p* < 0.05). The contents of hesperidin and neohesperidin were positively correlated with the DPPH result (*p* < 0.05). The contents of total flavonoids were positively correlated with the ABTS and FRAP results (*p* < 0.05), but the results of CAA were not significantly correlated with most flavonoids or coumarins (*p* > 0.05). The complex components of the fingered citron extract and the physiological factors such as the absorption and metabolism of some compounds by cells can influence or change the performance of the antioxidants, so evaluation of the antioxidant capacity of the fingered citron extract with a single method may have certain limitations. And the combination of chemical antioxidant methods and the CAA method becomes necessary when studying the antioxidant capacity of bioactive compounds or extracts.

Relevant studies have shown that flavonoids have good antioxidant activity. Huang et al. showed that quercetin 7-rhamnoside in *Hypericum japonicum* had strong scavenging ability on DPPH free radicals and ABTS cationic free radicals, and could alleviate the liver damage induced by H_2_O_2_ by down-regulating the production of MDA, increasing the content of glutathione (GSH) and catalase (CAT) activity in the liver [34]. Gur et al. showed that hesperidin could regulate paclitaxel-induced liver injury in rats by increasing the activity of antioxidant enzymes such as SOD and CAT [35]. Other flavonoids such as neohesperidin [36], naringin [37], vitexin, and diosmin [38] have also been demonstrated to show antioxidant activity assessed with the assays of ABTS, DPPH, ferric reducing activity, oxygen radical absorbance capacity, or CAA. Hu et al. showed that neohesperidin dihydrochalcone could alleviate the liver injury induced by CCl_4_ in mice by increasing the activities of catalase (CAT), total superoxide dismutase (T-SOD), glutathione peroxidase (GP-X), and GSH [39].

The correlation between the main bioactive compounds in fingered citron and their antioxidant activities was analyzed by Pearson correlation coefficient. The results showed that the total flavonoid content was positively correlated with the antioxidant capacity of fingered citron (*p* < 0.01). Diosmin, hesperidin, and neohesperidin were important contributors to the antioxidant activity of fingered citron. The three flavonoids showed high contents in fingered citron and all have two phenolic hydroxyl groups and one methoxy group attached to the basic structure. Although the content of limettin is much higher than that of diosmin, hesperidin, and neohesperidin, there is no significant correlation between limettin and the antioxidant activities, including the results of CAA, DPPH, ABTS, and FRAP, indicating limettin is not a major contributor to the antioxidant activity of fingered citron. Limettin is a typical coumarin compound and there is no phenolic hydroxyl group in its structure. So, the antioxidant activity is considered to be mostly attributed to the phenolic hydroxyl groups of flavonoids.

## 4. Conclusions

In this study, 21 coumarins and 21 flavonoids in fingered citron could be detected and were quantitatively analyzed based on UPLC-QqQ-MS/MS. The types of flavonoids and coumarins in different growth periods were basically the same, and the total contents of flavonoids and coumarins was in the order of November ˃ July ˃ September. Hesperidin and diosmin were the flavonoids with the highest contents, and limettin showed the highest content among coumarins. The fingered citron in different growth periods showed good antioxidant activity in vitro, which was positively correlated with the concentration. The oxidative stress model of RAW264.7 cells indicated that fingered citron extract can effectively reduce the contents of NO, MDA, and ROS in cells and increase the activity of SOD so as to effectively alleviate the damage to cells induced by H_2_O_2_. The regulatory activity of the extract from November on cellular oxidative stress was the highest among the three growth periods. There was a significantly positive correlation between the total flavonoid content and the antioxidant capacity of fingered citron (*p* < 0.01). Diosmin, hesperidin, and neohesperidin were the important flavonoids that contributed to the antioxidant activity of fingered citron. This study will provide a foundation for making use of the germplasm of fingered citron and for exploiting related products with nutritional and functional values.

## Figures and Tables

**Table 1:** APC index and ranking of antioxidant activity of fingered citron extracts at different growth periods of July (7J), September (9J), and November (11J).

	Concentration (mg/mL)	DPPH	ABTS	FRAP	APC Index	Rank
7J	3.00	47.2 ± 0.54 ^a^	157.21 ± 3.28 ^b^	59.42 ± 1.34 ^b^	91.06%	2
	6.00	84.98 ± 0.42 ^a^	306.79 ± 5.24 ^c^	105.8 ± 1.14 ^b^		
	12.00	115.2 ± 0.98 ^b^	508.88 ± 8.1 ^c^	183.62 ± 1.75 ^b^		
	18.00	147.76 ± 0.63 ^c^	677.63 ± 1.02 ^b^	267.83 ± 1.55 ^b^		
	24.00	187.09 ± 0.42 ^c^	778.04 ± 5.14 ^b^	339.42 ± 0.74 ^b^		
9J	3.00	39.31 ± 0.87 ^c^	158.88 ± 1.02 ^b^	46.81 ± 2.14 ^c^	88.38%	3
	6.00	67.42 ± 0.31 ^c^	318.88 ± 2.7 ^b^	102.17 ± 1.06 ^c^		
	12.00	106.42 ± 0.57 ^c^	537.21 ± 6.24 ^b^	178.41 ± 0.54 ^c^		
	18.00	153.53 ± 0.27 ^b^	693.46 ± 3.12 ^a^	256.96 ± 2.56 ^c^		
	24.00	189.53 ± 0.72 ^b^	789.29 ± 5.62 ^a^	336.38 ± 1.82 ^b^		
11J	3.00	41.31 ± 0.57 ^b^	173.46 ± 1.56 ^a^	63.48 ± 0.94 ^a^	100.00%	1
	6.00	81.53 ± 1.25 ^b^	359.71 ± 1.56 ^a^	103.91 ± 1.55 ^a^		
	12.00	140.09 ± 0.96 ^a^	586.79 ± 2.12 ^a^	200.87 ± 0.94 ^a^		
	18.00	193.09 ± 1.5 ^a^	691.38 ± 2.04 ^a^	285.51 ± 3.89 ^a^		
	24.00	229.53 ± 0.47 ^a^	794.71 ± 1.56 ^a^	383.33 ± 1.82 ^a^		

Note: lowercase letters in superscript indicate differences in antioxidant activity at different growth periods under the same concentration and the same method. The same lowercase letters mean no significant difference; different lowercase letters represent significant differences.

## Data Availability

The data presented in this study are available on request from the corresponding author. The data are not publicly available due to privacy.

## Supplementary Materials

The following supporting information can be downloaded at: https://www.mdpi.com/article/10.3390/foods14020180/s1, Figure S1: Effects of fingered citron extracts on the activity of RAW264.7 cells; Figure S2: Effects of H_2_O_2_ concentrations on RAW264.7 cells; Figure S3: Effects of different concentrations of H_2_O_2_ on survival rate of RAW264.7 cells; Figure S4: Effect of fingered citron extract on survival rate of RAW264.7 cells in the presence of H_2_O_2_. Different lowercase letters represent significant differences between groups (*p* < 0.05). Table S1: Information on commercial standards; Table S2: Reagents and materials for RAW264.7 cell experiments; Table S3: MRM parameters; Table S4: Information related to method validation; Table S5: Flavonoid and coumarin contents in Jinhua fingered citron.
