# Supplementary material for: Quantitative Analysis of Flavonoids and Coumarins from Fingered Citron in Different Growth Periods and Their Regulatory Effects on Oxidative Stress
Source: Foods. 2025 Jan 9;14(2):180. doi: 10.3390/foods14020180 (PMC11765496; doi:10.3390/foods14020180)
Supplement: Supplementary file 1 [file foods-14-00180-s001.zip › foods-3355849-supplementary.pdf]

Supplementary Material

for

Quantitative analysis of flavonoids and coumarins from fingered citron at different growth periods and their regulatory effects on oxidative stress

Tao Tan [a], Man Xu [a], Xianlong Hong [a], Zhenyuan Li [a], Jiangnan Li [b], Bining Jiao [b], Xijuan Zhao [a,b*]

[a] *College of Horticulture and Landscape Architecture, Southwest University, Chongqing, China*

[b] *Key Laboratory of Quality and Safety Control for Citrus Fruits, Ministry of Agriculture and Rural Affairs, Southwest University, Chongqing, China*

Correspondence:

Xijuan Zhao (corresponding author): xijuanzh@swu.edu.cn, 86-23-68250483

Table S1 Information of commercial standards.

Table S2 Reagents and materials for RAW264.7 cell experiments.

Table S3 MRM parameters.

Table S4 Information related to method validation.

Table S5 Flavonoid and coumarin contents in Jinhua fingered citron.

Figure S1 Effects of fingered citron extracts on the activity of RAW264.7 cells.

Figure S2 Effects of H_2O_2 concentrations on RAW264.7 cells.

Figure S3 Effects of different concentrations of H_2O_2 on survival rate of RAW264.7 cells.

Figure S4 Effect of fingered citron extract on survival rate of RAW264.7 cells in the presence of H_2O_2. Different lowercase letters represent significant differences between groups ($P < 0.05$).

Table S1 Information of commercial standards

Compound name	CAS No.	Chemical formula	Purity	Supplier
4',5,6,7-Tetramethoxyflavone	1168-42-9	$C_{19}H_{18}O_6$	≥95%	Shanghai yuanye Bio-Technology Co., Ltd. (Shanghai, China)
5-Geranyloxy-7-methoxycoumarin	7380-39-4	$C_{20}H_{24}O_4$	≥99%	ChromaDex (USA)
6',7'-Dihydroxybergamottin	145414-76-2	$C_{21}H_{24}O_6$	≥97%	Sigma-Aldrich (USA)
6',7'-Epoxybergamottin	206978-14-5	$C_{21}H_{22}O_5$	≥95%	Sigma-Aldrich (USA)
Cnidilin	1434 8-22-2	$C_{17}H_{16}O_5$	≥98%	Shanghai yuanye Bio-Technology Co., Ltd. (Shanghai, China)
Byakangelicol	26091-79-2	$C_{17}H_{16}O_6$	≥98%	Chengdu Chroma-Biotechnology Co., Ltd. (Chengdu, China)
Byakangelicin	19573-01-4	$C_{17}H_{18}O_7$	≥98%	Shanghai yuanye Bio-Technology Co., Ltd. (Shanghai, China)
Scoparone	120-08-1	$C_{11}H_{10}O_4$	≥98%	Jindi Technology.co., Ltd (Beijing, China)
Psoralen	66-97-7	$C_{11}H_6O_3$	≥98%	Jindi Technology.co., Ltd (Beijing, China)
Auraptene	495-02-3	$C_{19}H_{22}O_3$	≥98%	Sigma-Aldrich (USA)
Scopoletin	92-61-5	$C_{10}H_8O_4$	≥98%	Chengdu Chroma-Biotechnology Co., Ltd. (Chengdu, China)
Bergapten	484-20-8	$C_{12}H_8O_4$	≥98%	Jindi Technology.co., Ltd (Beijing, China)
Phloretin	60-82-2	$C_{15}H_{14}O_5$	≥98%	Anpel Laboratory Technologies (Shanghai) Inc. (Shanghai, China)
Tangeretin	481-53-8	$C_{20}H_{20}O_7$	≥98%	Anpel Laboratory Technologies (Shanghai) Inc. (Shanghai, China)
Vitexicarpin	479-91-4	$C_{19}H_{18}O_8$	≥98%	Chengdu Chroma-Biotechnology Co., Ltd. (Chengdu, China)

Compound name	CAS No.	Chemical formula	Purity	Supplier
Vitexin	3681-93-4	$C_{21}H_{20}O_{10}$	≥98%	Shanghai yuanye Bio-Technology Co., Ltd. (Shanghai, China)
Limettin	487-06-9	$C_{11}H_{10}O_4$	≥98%	Jindi Technology.co., Ltd (Beijing, China)
Imperatorin	482-44-0	$C_{16}H_{14}O_4$	≥98%	Jindi Technology.co., Ltd (Beijing, China)
Umbelliferone	93-35-6	$C_9H_6O_3$	≥98%	Jindi Technology.co., Ltd (Beijing, China)
Phellopterin	2543-94-4	$C_{17}H_{16}O_5$	≥98%	Shanghai yuanye Bio-Technology Co., Ltd. (Shanghai, China)
Osthole	484-12-8	$C_{15}H_{16}O_3$	≥98%	Shanghai yuanye Bio-Technology Co., Ltd. (Shanghai, China)
Oxypeucedanin hydrate	2643-85-8	$C_{16}H_{16}O_6$	≥98%	Shanghai yuanye Bio-Technology Co., Ltd. (Shanghai, China)
Narcissoside	604-80-8	$C_{28}H_{32}O_{16}$	≥98%	Shanghai yuanye Bio-Technology Co., Ltd. (Shanghai, China)
Sinensetin	2306-27-6	$C_{20}H_{20}O_7$	≥98%	Anpel Laboratory Technologies (Shanghai) Inc. (Shanghai, China)
Bergaptol	486-60-2	$C_{11}H_6O_4$	≥98%	Jindi Technology.co., Ltd (Beijing, China)
Diosmetin	520-34-3	$C_{16}H_{12}O_6$	≥98%	Chengdu Chroma-Biotechnology Co., Ltd. (Chengdu, China)
Vicenin-2	23666-13-9	$C_{27}H_{30}O_{15}$	≥98%	Chengdu Chroma-Biotechnology Co., Ltd. (Chengdu, China)
Oxypeucedanin	737-52-0	$C_{16}H_{14}O_5$	≥98%	Shanghai yuanye Bio-Technology Co., Ltd. (Shanghai, China)
Rhoifolin	17306-46-6	$C_{27}H_{30}O_{14}$	≥98%	Anpel Laboratory Technologies (Shanghai) Inc. (Shanghai, China)
Isomeranzin	1088-17-1	$C_{15}H_{16}O_4$	≥98%	Shanghai yuanye Bio-Technology Co., Ltd. (Shanghai, China)

Compound name	CAS No.	Chemical formula	Purity	Supplier
Isoimperatorin	482-45-1	$C_{16}H_{14}O_4$	≥99%	Shanghai yuanye Bio-Technology Co., Ltd. (Shanghai, China)
Marmesin	13849-08-6	$C_{14}H_{14}O_4$	≥98%	Shanghai yuanye Bio-Technology Co., Ltd. (Shanghai, China)
Hesperetin	520-33-2	$C_{16}H_{14}O_6$	≥98%	Anpel Laboratory Technologies (Shanghai) Inc. (Shanghai, China)
Hesperidin	520-26-3	$C_{28}H_{34}O_{15}$	≥97%	Anpel Laboratory Technologies (Shanghai) Inc. (Shanghai, China)
Diosmin	520-27-4	$C_{28}H_{32}O_{15}$	≥98%	Chengdu Chroma-Biotechnology Co., Ltd. (Chengdu, China)
Methyl hesperidin	11013-97-1	$C_{29}H_{36}O_{15}$	≥98%	Chengdu Chroma-Biotechnology Co., Ltd. (Chengdu, China)
Rutin	153-18-4	$C_{27}H_{30}O_{16}$	≥99%	Anpel Laboratory Technologies (Shanghai) Inc. (Shanghai, China)
Luteolin	491-70-3	$C_{15}H_{10}O_6$	≥98%	Chengdu Chroma-Biotechnology Co., Ltd. (Chengdu, China)
Didymin	14259-47-3	$C_{28}H_{34}O_{14}$	≥99%	Anpel Laboratory Technologies (Shanghai) Inc. (Shanghai, China)
Neohesperidin	13241-33-3	$C_{28}H_{34}O_{15}$	≥98%	Anpel Laboratory Technologies (Shanghai) Inc. (Shanghai, China)
Isovitexin	29702-25-8	$C_{21}H_{20}O_{10}$	≥98%	Shanghai yuanye Bio-Technology Co., Ltd. (Shanghai, China)
Isorhamnetin	480-19-3	$C_{16}H_{12}O_7$	≥98%	Chengdu Chroma-Biotechnology Co., Ltd. (Chengdu, China)
Isoquercetin	21637-25-2	$C_{21}H_{20}O_{12}$	≥98%	Shanghai yuanye Bio-Technology Co., Ltd. (Shanghai, China)

Compound name	CAS No.	Chemical formula	Purity	Supplier
Narirutin	14259-46-2	$C_{27}H_{32}O_{14}$	≥99%	Shanghai yuanye Bio-Technology Co., Ltd. (Shanghai, China)
Naringenin	480-41-1	$C_{15}H_{12}O_5$	≥98%	Chengdu Chroma-Biotechnology Co., Ltd. (Chengdu, China)
Naringin	10236-47-2	$C_{27}H_{32}O_{14}$	≥98%	Anpel Laboratory Technologies (Shanghai) Inc. (Shanghai, China)
Poncirin	14941-08-3	$C_{28}H_{34}O_{14}$	≥98%	Anpel Laboratory Technologies (Shanghai) Inc. (Shanghai, China)
Quercetin	117-39-5	$C_{15}H_{10}O_7$	≥98%	Shanghai yuanye Bio-Technology Co., Ltd. (Shanghai, China)

Table S2 Reagents and materials for RAW264.7 cell experiments

Reagent and material names	Supplier
RAW264.7 cell	Wuhan Pricella Biotechnology Co., Ltd. (Wuhan, China)
Phosphate buffered saline (PBS)	Beijing Labgic Technology Co., Ltd. (Beijing, China)
Dulbecco's modified eagle medium (DMEM)	Beijing Solarbio Science & Technology Co., Ltd (Beijing, China)
Fetal bovine serum(FBS)	Yeasen Biotechnology (Shanghai) Co. Ltd (Shanghai, China)
Penicillin - streptomycin	Yeasen Biotechnology (Shanghai) Co. Ltd (Shanghai, China)
Dimethyl sulfoxide(DMSO)	Shanghai Xingke Solvents Co.,Ltd. (Shanghai, China)
Methanol	Chengdu Chroma-Biotechnology Co., Ltd. (Chengdu, China)
Ethyl alcohol	Chongqing Chuandong Chemical(Group)Co.,Ltd. (Chongqing, China)
6/96 Well cell culture plate	Beijing Labgic Technology Co., Ltd (Beijing, China)
Cell scraper	Beijing Labgic Technology Co., Ltd (Beijing, China)
Hemocytometer	Shanghai Qiujing Biochemical Reagent Instrument Co.,Ltd. (Shanghai, China)
Cell Counting Kit (CCK-8)	Yeasen Biotechnology (Shanghai) Co. Ltd (Shanghai, China)
Nitrite Assay Kit (Griess Kit)	Beijing Leagene Biotechnology Co., Ltd. (Beijing, China)
Reactive oxygen species (ROS) detection kit	Beijing Solarbio Science & Technology Co., Ltd (Beijing, China)
Malondialdehyde (MDA) content detection kit	Beijing Solarbio Science & Technology Co., Ltd (Beijing, China)
Superoxide dismutase (SOD) activity test kit	Beijing Solarbio Science & Technology Co., Ltd (Beijing, China)

Table S3 MRM parameters

Compound name	Ion species	Precursor ion (*m*/*z*)	Product ion 1 (*m*/*z*)	CE (V)	Product ion 2 (*m*/*z*)	CE (V)
4',5,6,7-Tetramethoxyflavone	$[M+H]^+$	343.1	313.4	-28	282.4	-25
5-Geranyloxy-7-methoxycoumarin	$[M+H]^+$	329.3	137.2	-34	193.1	-16
6',7'-Dihydroxybergamottin	$[M+Na]^+$	395.2	194.2	-21	225.1	-20
6',7'-Epoxybergamottin	$[M+H]^+$	355.1	202.8	-18	153.1	-11
Cnidilin	$[M+H]^+$	301	233.2	-13	217.1	-15
Byakangelicol	$[M+H]^+$	317	233.2	-14	231.2	-19
Byakangelicin	$[M+H]^+$	356.9	253.9	-20	239.1	-27
Scoparone	$[M+H]^+$	207.1	151	-20	191.3	-23
Psoralen	$[M+H]^+$	187	115.2	-21	131.1	-24
Auraptene	$[M+H]^+$	299.1	163.1	-12	137.2	-8
Scopoletin	$[M+H]^+$	193.25	133.2	-21	122.25	-28
Bergapten	$[M+H]^+$	217.1	174	-26	202	-21
Phloretin	$[M+H]^+$	275.1	107.1	-22	168.9	-11
Tangeretin	$[M+H]^+$	373.1	343	-26	358.1	-19
Vitexicarpin	$[M+H]^+$	375.1	342.2	-26	359.1	-26
Vitexin	$[M+H]^+$	433.1	397.4	-22	313.1	-28
Limettin	$[M+H]^+$	207.1	164	-21	191.9	-19
Imperatorin	$[M+H]^+$	271.1	203.1	-11	147.1	-34
Umbelliferone	$[M+H]^+$	163.2	106.8	-22	118.9	-18
Phellopterin	$[M+H]^+$	301.2	245.3	-15	233.2	-13
Osthole	$[M+H]^+$	245	189	-13	131.1	-26
Oxypeucedanin hydrate	$[M+H]^+$	305.5	149.2	-23	203.3	-23
Narcissoside	$[M+H]^+$	625.2	317.1	-24	479	-39
Sinensetin	$[M+H]^+$	373.1	343.3	-30	312.1	-24
Bergaptol	$[M+H]^+$	203.1	147.1	-22	131.1	-22

Compound name	Ion species	Precursor ion (*m*/*z*)	Product ion 1 (*m*/*z*)	CE (V)	Product ion 2 (*m*/*z*)	CE (V)
Diosmetin	$[M+H]^+$	301.1	286.1	-26	258	-36
Vicenin-2	$[M+H]^+$	595.2	457.3	-20	577.1	-14
Oxypeucedanin	$[M+H]^+$	287	203.3	-17	85	-19
Rhoifolin	$[M+H]^+$	579.1	270.9	-20	433.2	-19
Isomeranzin	$[M+H]^+$	261.1	189.1	-17	243.2	-11
Isoimperatorin	$[M+H]^+$	271.1	147.1	-30	203.1	-12
Marmesin	$[M+H]^+$	247	229.3	-19	175.3	-23
Hesperetin	$[M-H]^-$	301	164.2	25	150.9	22
Hesperidin	$[M-H]^-$	609.1	301.1	26	286	44
Diosmin	$[M-H]^-$	607.2	284.1	49	299.1	28
Methyl hesperidin	$[M-H]^-$	623.2	315.2	30	356.6	40
Rutin	$[M-H]^-$	609.1	300	40	271	51
Luteolin	$[M-H]^-$	285	174.9	24	133	34
Didymin	$[M-H]^-$	593.1	285.1	28	327.1	26
Neohesperidin	$[M-H]^-$	609.1	286	40	301.1	36
Isovitexin	$[M-H]^-$	431	340.9	18	311.2	23
Isorhamnetin	$[M-H]^-$	315	300.1	21	150.8	27
Isoquercetin	$[M-H]^-$	463	300.2	27	270.9	47
Narirutin	$[M-H]^-$	579.2	313.2	27	150.9	44
Naringenin	$[M-H]^-$	271	151	18	119	25
Naringin	$[M-H]^-$	579.2	150.9	40	313.2	28
Poncirin	$[M-H]^-$	593.1	327.1	25	285.1	31
Quercetin	$[M-H]^-$	301.1	151.3	22	179.25	18

Note: desolvation temperature, 650 °C; DL temperature, 250 °C; interface temperature, 400 °C; CID gas, 270 kPa; interface voltage, 1.0 kV; detector voltage, 2.16 kV; heating gas flow, 15 L/min; dry gas flow, 3L/min.

Table S4 Information related to method validation

Number	Compound name	Time（min）	Regression equation	r^2	Calibration range（μg/L）	LODs（μg/L）	LOQs（μg/L）	Recovery 1	Recovery 2
1	4',5,6,7-Tetramethoxyflavone	9.26	f(x)=91830.0x+166474	0.9983	10-400	0.04	0.12	101.10%	95.46%
2	5-Geranyloxy-7-methoxycoumarin	12.26	f(x)=52450.0x+157.859	0.9968	0.1-400	0.09	0.27	87.11%	99.16%
3	6',7'-Dihydroxybergamottin	9.02	f(x)=760.774x+7078.69	0.9971	10-400	0.92	2.8	81.11%	103.23%
4	6',7'-Epoxybergamottin	10.34	f(x)=2044.05x+968.721	0.9979	0.1-400	0.34	1.04	106.47%	107.79%
5	Cnidilin	10.19	f(x)=156242x+7612.69	0.9982	0.1-400	0.02	0.05	116.68%	119.03%
6	Byakangelicol	9.21	f(x)=88283.8x+11649.0	0.9977	0.1-400	0.03	0.09	77.27%	125.91%
7	Byakangelicin	7.81	f(x)=1953.23x+9865.57	0.9958	5-400	1.04	3.16	76.23%	103.14%
8	Scoparone	6.78	f(x)=20043.0x-2482.20	0.9972	1-400	1.11	3.35	123.52%	80.77%
9	Psoralen	8.30	f(x)=23903.2x+75655.9	0.9979	10-400	0.64	1.94	106.31%	89.09%
10	Auraptene	11.93	f(x)=33812.0x+20290.2	0.9966	0.1-400	0.11	0.35	96.64%	108.00%
11	Scopoletin	5.36	f(x)=31975.6x+97175.1	0.9956	10-400	1.6	4.84	123.53%	95.54%
12	Bergapten	8.83	f(x)=31435.7x-2595.51	0.9993	1-400	0.19	0.57	108.60%	112.03%
13	Phloretin	8.17	f(x)=23578.7x+4519.35	0.9996	0.1-400	0.34	1.04	120.18%	122.81%
14	Tangeretin	9.55	f(x)=168809x+986993	0.9909	10-400	0.02	0.05	92.82%	100.93%
15	Vitexicarpin	9.19	f(x)=11764.4x+1674.77	0.9983	0.1-400	0.01	0.02	121.41%	120.46%
16	Vitexin	5.12	f(x)=18454.2x+154985	0.9984	10-400	0.16	0.47	93.54%	84.45%
17	Limettin	8.72	f(x)=15832.9x+6468.71	0.9971	5-400	0.17	0.51	97.05%	86.86%
18	Imperatorin	9.82	f(x)=3755.92x+22956.7	0.9990	10-400	0.74	2.23	110.14%	102.98%
19	Umbelliferone	5.23	f(x)=1088.28x+6414.51	0.9983	25-400	2.65	8.02	116.60%	113.29%
20	Phellopterin	10.04	f(x)=33095.1x+67366.6	0.9993	5-400	0.1	0.3	107.75%	100.05%

Number	Compound name	Time (min)	Regression equation	r^2	Calibration range (μg/L)	LODs (μg/L)	LOQs (μg/L)	Recovery 1	Recovery 2
21	Osthole	10.10	f(x)=119114x+40344.3	0.9992	1-400	0.02	0.05	115.23%	113.21%
22	Oxypeucedanin hydrate	7.61	f(x)=3934.90x+97213.8	0.9953	10-400	0.28	0.83	97.32%	113.48%
23	Narcissoside	5.67	f(x)=523.322x+3255.36	0.9939	25-400	0.3	0.89	116.21%	107.50%
24	Sinensetin	8.97	f(x)=35582.6x+152228	0.9973	5-400	0.16	0.48	113.30%	104.12%
25	Bergaptol	7.61	f(x)=670.367x+8179.08	0.9998	5-400	4.46	13.52	112.49%	91.91%
26	Diosmetin	8.39	f(x)=33338.6x+28634.5	0.9995	1-400	0.06	0.18	115.60%	105.28%
27	Vicenin-2	4.03	f(x)=1294.45x+11426.4	0.9948	10-300	0.01	0.04	84.27%	91.07%
28	Oxypeucedanin	9.24	f(x)=46117.0x+10075.6	0.9974	1-400	0.03	0.1	96.40%	113.67%
29	Rhoifolin	5.82	f(x)=2503.28x-1547.16	0.9973	1-400	0.12	0.37	81.43%	95.78%
30	Isomeranzin	8.99	f(x)=200065x+139976	0.9946	5-300	0.02	0.06	112.66%	89.45%
31	Isoimperatorin	10.26	f(x)=28113.7x+19520.9	0.9976	1-400	0.13	0.4	114.42%	101.16%
32	Marmesin	7.28	f(x)=23081.8x+98385.2	0.9962	10-400	0.2	0.62	97.06%	91.11%
33	Hesperetin	8.41	f(x)=4386.55x+7221.74	0.9989	1-400	0.08	0.26	86.03%	107.56%
34	Hesperidin	5.99	f(x)=1521.51x+11706.0	0.9976	10-400	0.05	0.14	119.95%	114.65%
35	Diosmin	5.94	f(x)=2604.01x+4663.85	0.9969	5-400	0.02	0.07	92.07%	106.40%
36	Methyl hesperidin	6.69	f(x)=1722.97x+636.541	0.9970	5-400	0.89	2.69	107.45%	94.66%
37	Rutin	5.05	f(x)=445.427x+115.037	0.9972	5-400	0.06	0.17	86.47%	99.94%
38	Luteolin	7.50	f(x)=7899.98x-638.057	0.9976	0.1-400	0.18	0.05	100.65%	100.52%
39	Didymin	7.35	f(x)=2102.23x-3233.42	0.9932	5-400	1.31	3.97	117.69%	106.44%
40	Neohesperidin	6.19	f(x)=817.847x+122.552	0.9932	5-400	2.19	6.65	105.46%	92.34%
41	Isovitexin	5.13	f(x)=7419.26x+4070.90	0.9989	1-400	0.26	0.79	112.90%	109.64%
42	Isorhamnetin	8.43	f(x)=13299.6x+569.936	0.9992	0.1-400	0.02	0.06	95.61%	96.92%
43	Isoquercetin	5.28	f(x)=4359.64x+7091.96	0.9947	5-400	0.15	0.46	96.31%	116.60%
44	Narirutin	5.60	f(x)=446.944x-1238.82	0.9961	5-400	1.17	3.55	119.83%	102.86%
45	Naringenin	8.19	f(x)=8075.62x-586.651	0.9936	1-400	0.18	0.56	117.69%	93.40%

Number	Compound name	Time（min）	Regression equation	r^2	Calibration range（μg/L）	LODs（μg/L）	LOQs（μg/L）	Recovery 1	Recovery 2
46	Naringin	5.81	f(x)=464.686x+121.332	0.9927	1-400	0.08	0.23	116.55%	103.96%
47	Poncirin	7.57	f(x)=2104.65x+790.121	0.9985	5-400	0.42	1.27	119.19%	93.98%
48	Quercetin	7.58	f(x)=4890.74x-16944.9	0.9988	5-400	1.37	4.15	101.11%	101.61%

Table S5 Flavonoid and coumarin contents in Jinhua fingered citron

	7J（μg/g）	9J（μg/g）	11J（μg/g）
Hesperidin	627.71±26.41	639.45±56.37	974.29±67.94
Diosmin	790.09±25.71	749.21±26.77	989.36±30.26
Rutin	308.41±30.69	203.76±17.8	232.18±13.62
Narcissoside	81.63±2.39	117.36±3.81	74.55±4.19
Neohesperidin	34.86±2.01	33.04±1.17	55.83±4.92
Vicenin-2	15.23±0.27	28.06±0.59	19.69±1.72
Narirutin	3.16±0.35	2.12±0.3	2.84±0.43
Isovitexin	2.27±0.06	1.55±0.06	1.75±0.09
Naringin	0.48±0.09	2.39±0.08	3.18±0.2
Methyl hesperidin	1.2±0.08	1.1±0.05	1.68±0.18
Vitexicarpin	0.59±0.09	1.46±0.2	1.15±0.05
Didymin	0.38±0.04	1.12±0.11	0.9±0.06
Isoquercetin	1.47±0.16	0.02±0.002	0.01±0.001
Tangeretin	0.28±0.02	0.62±0.08	0.53±0.02
Hesperetin	0.11±0.01	0.31±0.08	0.06±0.01
Sinensetin	0.04±0.01	1.52±0.03	0.64±0.01
Naringenin	0.03±0.002	0.01±0.002	0.01±0.001
Luteolin	0.01±0.001	0.01±0.002	0.01±0.001
Diosmetin	0.29±0.02	0.61±0.13	0.13±0.01
Isorhamnetin	ND	ND	ND
Quercetin	ND	ND	ND
Rhoifolin	ND	ND	ND
4',5,6,7-Tetramethoxyflavone	NQ	NQ	NQ
Phloretin	ND	ND	ND
Vitexin	2.06±0.03	1.55±0.1	2.1±0.04
Poncirin	ND	ND	ND
Limettin	1721.3±172.6	1513.28±146.11	2078.92±75.44
Oxypeucedanin	590.34±16.08	123.33±1.65	389.22±5.56
Byakangelicol	229.21±6.18	137.1±12.37	221.4±3.75
Bergaptol	1.49±1.49	0.58±0.58	0.03±0.03
Byakangelicin	0.12±0.12	1.06±1.06	0.56±0.56
Oxypeucedanin hydrate	26.16±1.07	9.36±0.6	12.97±0.95
Scopoletin	12.44±1.08	10.38±0.37	2.72±0.12
Bergapten	5.65±0.51	0.93±0.01	1.48±0.03
Phellopterin	1.71±0.01	1±0.17	0.87±0.06
Imperatorin	3.2±0.04	0.15±0.01	0.72±0.05
Umbelliferone	1.78±0.22	2.41±0.14	0.69±0.05
Scoparone	0.36±0.004	0.89±0.04	0.18±0.02
Marmesin	0.27±0.02	0.66±0.04	0.21±0.01
Isoimperatorin	0.35±0.02	0.24±0.11	0.09±0.001
Auraptene	0.06±0.002	0.02±0.0005	0.03±0.001

	7J（μg/g）	9J（μg/g）	11J（μg/g）
Cnidilin	0.01±0.0002	0.01±0.01	0.01±0.001
Isomeranzin	0.04±0.01	0.07±0.01	0.09±0.01
5-Geranyloxy-7-methoxycoumarin	0.0004±0.00009	0.002±0.00002	ND
6',7'-Dihydroxybergamottin	NQ	0.02±0.01	0.24±0.03
6',7'-Epoxybergamottin	0.002±0.001	NQ	ND
Psoralen	ND	ND	ND
Osthole	NQ	NQ	NQ

NQ, detected but not quantified. ND, not detected.

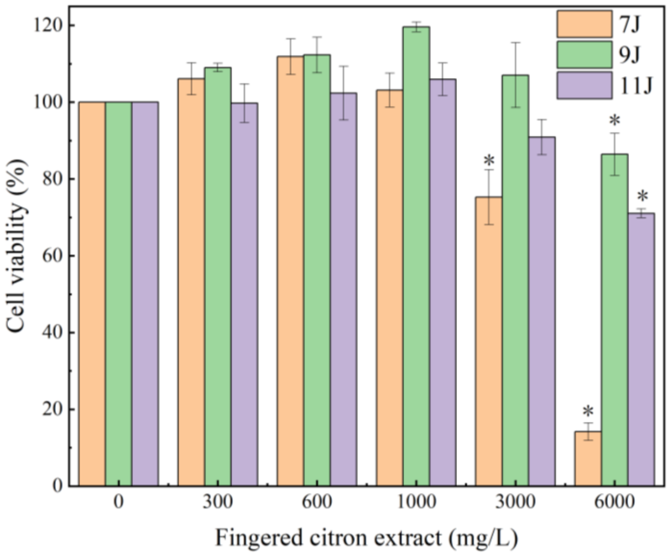

Figure S1 Effects of fingered citron extracts collected at July (7J), September (9J) and November (11J) on the activity of RAW264.7 cells.

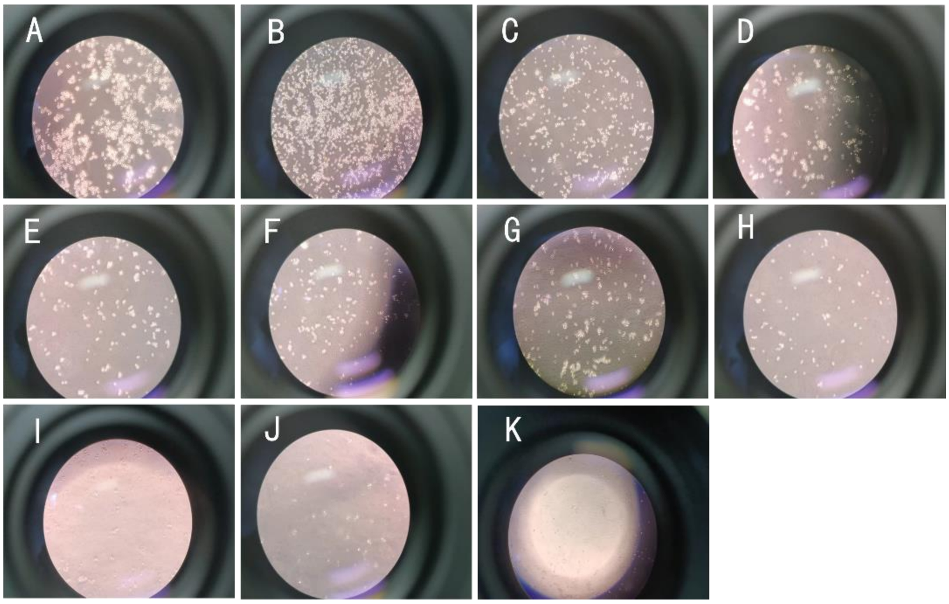

Figure S2 Effects of different H_2O_2 concentrations on RAW264.7 cells
(A) RAW264.7 cells without H_2O_2 as control group; (B-K) RAW264.7 cells treated with 0.001%-0.01% H_2O_2 in turn.

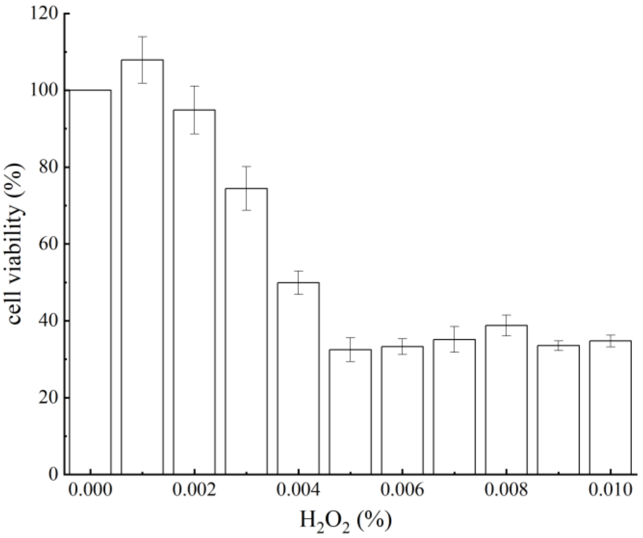

Figure S3 Effects of different concentrations of H_2O_2 on survival rate of RAW264.7 cells.

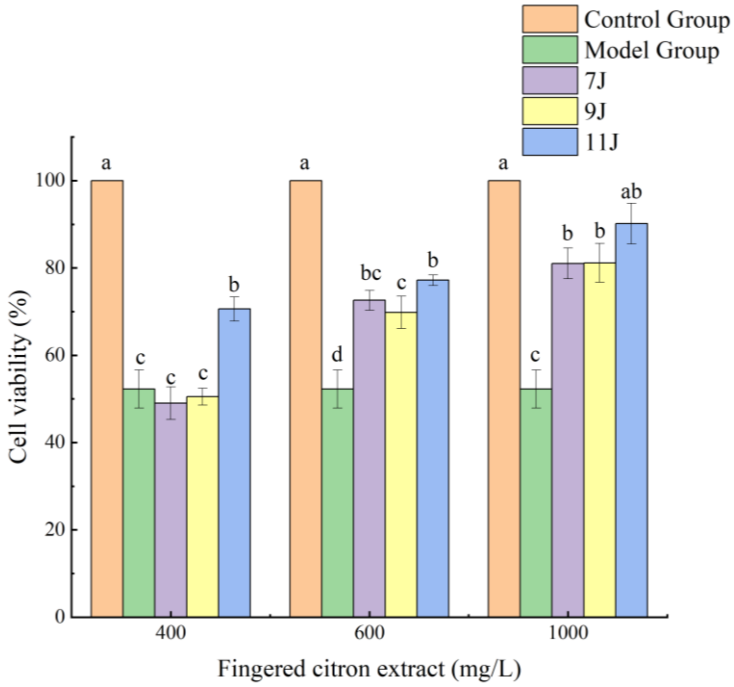

Figure S4 Effect of fingered citron extract on survival rate of RAW264.7 cells in the presence of H_2O_2. Different lowercase letters represent significant differences between groups ($P < 0.05$).
